# Gastrointestinal involvement in systemic sclerosis: An updated review

**DOI:** 10.1097/MD.0000000000031780

**Published:** 2022-11-11

**Authors:** Mahmoud Nassar, Victoria Ghernautan, Nso Nso, Akwe Nyabera, Francisco Cuevas Castillo, Wan Tu, Luis Medina, Camelia Ciobanu, Mostafa Alfishawy, Vincent Rizzo, Saphwat Eskaros, Mamdouh Mahdi, Mohamed Khalifa, Mohamed El-Kassas

**Affiliations:** a Department of Medicine, Icahn School of Medicine at Mount Sinai, NYC Health+Hospitals, Queens, NY, USA; b Internal Medicine, Saint Barnabas Hospital, Bronx, NY, USA; c Internal Medicine Department, Faculty of Medicine, Cairo University, Cairo, Egypt; d Division of Gastroenterology, Department of Medicine, Icahn School of Medicine at Mount Sinai, NYC Health+Hospitals, Queens, NY, USA; e Internal Medicine Department, Faculty of Medicine, Helwan University, Cairo, Egypt; f Hospital Management Department, Helwan University, Cairo, Egypt; g Endemic Medicine Department, Faculty of Medicine, Helwan University, Cairo, Egypt.

**Keywords:** gastrointestinal, scleroderma, systemic sclerosis

## Abstract

The gastrointestinal tract (GI) is the second most affected organ system in individuals suffering from systemic/localized scleroderma (SSc) or localized scleroderma. SSc can affect any part of the GI, between the oral cavity and anorectum. The annual incidence of SSc in the United States is estimated to be 19.3 cases per million adults, with the highest incidence reported in people aged 44 to 55. Females are 5 times more likely than males to suffer from SSc. Morbidity and mortality rates associated with SSc are predominantly elevated among patients with GI manifestations. Esophageal and intestinal manifestations impact 90% and 40% to 70% of patients with systemic scleroderma, respectively. SSc patients are known to suffer from small bowel hypomotility and small intestinal bacterial overgrowth, which cause malabsorption and malnutrition, ultimately contributing to the 50% mortality rate. Fecal incontinence is a common symptom of SSc that can lead to depression. SSc patients may suffer from gastrointestinal complications that can negatively impact their quality of life on a daily basis. Multidisciplinary approaches are necessary for systematically managing gastrointestinal complications associated with SSc. A prospective study should focus on developing targeted therapies to improve recovery patterns and prognosis in cases of SSc. This article summarizes the epidemiology, commonly reported clinical manifestations, complications, and available treatments for treating GI pathology in SSc patients.

## 1. Introduction

A systemic/localized scleroderma (SSc) is characterized by generalized abnormalities of the small arteries, micro-vessels, and connective tissues, with an annual incidence of 19.3 cases (per million adults) in the United States.^[1,2]^ SSc is most prevalent in individuals between the ages of 44 and 55. Women have a higher incidence of SSc than men, with an increased prevalence among African Americans.^[2]^ In more than 90% of cases, this progressive autoimmune condition is accompanied by organ fibrosis, microvascular complications, and gastrointestinal complications.^[3–5]^

Patients with SSc often report signs of inflammation, skin fibrosis, vascular abnormalities, organ deterioration, and increased concentration of autoantibodies.^[6]^ The SSc also impacts the physiological functioning of the heart, lungs, kidneys, and digestive tract. The cohort study by Jaeger et al (2016) affirmed the simultaneous onset of 50% of incidental organ manifestations in SSc cases within 2 years after the onset of Raynaud’s phenomenon.^[7]^ The gastrointestinal complications in diffuse or limited scleroderma adversely impact the functioning of visceral organs.^[1,8]^ These manifestations emanate from the impact of gastrointestinal tract (GI) fibrosis, vascular damage, and inflammatory processes.^[1]^ The case-control study by Ciaula et al (2008) revealed the impact of dyspepsia on diffuse gastrointestinal dysmotility and gastric antrum distension restriction in SSc scenarios.^[2]^ The EULAR Scleroderma Trial and Research cohort revealed 6.6% of deaths from SSc that resulted from GI complications among elderly patients and patients with diffuse skin involvement.^[7,9,10]^ The affected patients developed a high frequency of GI symptoms at an early stage of systemic scleroderma.

SSc manifestations’ frequency, intensity, and severity vary with the pathology in the anorectum, esophagus, and other gastrointestinal locations.^[6,8]^ Increased collagen deposition and other extracellular matrix components lead to fibrotic changes in the upper and lower GI tract, resulting in dysmotility, malabsorption, and dilation of the intestine.^[11]^ The scleroderma symptoms commonly include meteorism, dysmotility of the esophagus, heartburn, dysphagia, nausea, vomiting, diarrhea, and constipation.^[6,7,11]^ Schmeiser et al (2012) found that amongst 90 patients with SSc, approximately 98.9% suffered from GI symptoms regardless of the stage of the disease.^[11]^ The commonly reported symptoms of SSc included meteorism (87.8%) and fecal incontinence (23.5%). Using a large-scale nationwide database, Lin et al (2019) revealed that SSc patients exhibited a high risk of GI bleeding across gastrointestinal locations affected with peptic and non-peptic ulcers. Their findings also revealed the GI bleeding predisposition of the systematic scleroderma male patients with diabetes mellitus, hypertension, dependence on steroids, antiplatelets, and non-steroidal anti-inflammatory drugs.^[10]^ The SSc increases the risk for fatal manifestations, including Ogilvie syndrome or intestinal pseudo-obstruction.^[11]^ GI symptoms of 5.3% to 55.6% of SSc patients reportedly increase their risk of malnutrition.^[12]^

The heterogeneity of symptoms in SSc cases often masks the risk assessment interventions. The diagnostic challenges in SSC scenarios eventually barricade assessing the patient’s predisposition for severe and progressive gastrointestinal disease. The heterogeneity of manifestations also restricts their correlation with immune-mediated disease activity versus the reported clinical deterioration. The diagnostic difficulties eventually challenge the medical decisions based on the administration of immunosuppressants, GI medications, and promotility agents in SSc scenarios.^[8]^ Additionally, increased symptom burden secondary to GI dysautonomia has been associated with higher levels of emotional distress. Studies have shown that when asked to complete the PHQ-9 Patient Health Questionnaire, depression was 25% higher in SSc patients than in the age-adjusted healthy general population. When addressing patient needs, physical and emotional aspects must be considered to optimize the patient care processes.^[13]^

## 2. Morbidity and mortality of gastrointestinal involvement in SSc

The standard mortality ratio of SSc is 3 to 4 per million per year.^[14]^ The cumulative death rates of 13.5%, 25.1%, 37.5%, and 58.2% were reportedly recorded after the first, 5, 10, and 20 years of SSc diagnosis.^[15]^ Severe GI complications exist in 8% of SSc patients.^[16]^ GI tract complications are the 4th most common cause of mortality in 7.6% of SSc scenarios. Other significant causes of SSc are attributed to potential complications in the lung (47.8%), heart (25.6%), and kidney (18.5%).^[15]^ Approximately 8.8% and 9.7% of all-cause Mortality in SSc are attributed to gastrointestinal manifestations within 5 and 10 years of diagnostic affirmation.^[17]^

The high prevalence of GI symptoms in scleroderma scenarios substantially increases the comorbidity and mortality of the affected patients. A recent study by Thoua et al (2010) revealed 97% of SSc patients with upper (94%) and lower (79%) GI symptoms.^[3]^ 50% to 90% of SSc patients exhibit esophageal dysfunction in various clinical scenarios.^[18]^ However, scleroderma exhibits the potential to impact any part of the GI.^[19]^ Steen et al (2000) revealed severe GI tract involvement in 4% and 8% of SSc patients at 3 and 9 years after diagnostic affirmation.^[17]^ The malabsorption in SSc scenarios leads to 50% mortality after 8.5 years of diagnostic confirmation.^[20]^ It also leads to small bowel pseudo-obstruction, leading to in-hospital Mortality and Mortality in 7.3% to 16% and 20% to 40% of the affected patients.^[16]^ Serious gastrointestinal disorders can lead to death, such as aspiration pneumonia caused by severe gastroesophageal reflux disease (GERD) or sepsis caused by an infection of the central line in patients receiving parenteral nutrition, thus underestimating the GI mortality rate.^[21]^

## 3. Brief pathophysiological concepts in SSc

**Figure 1.**
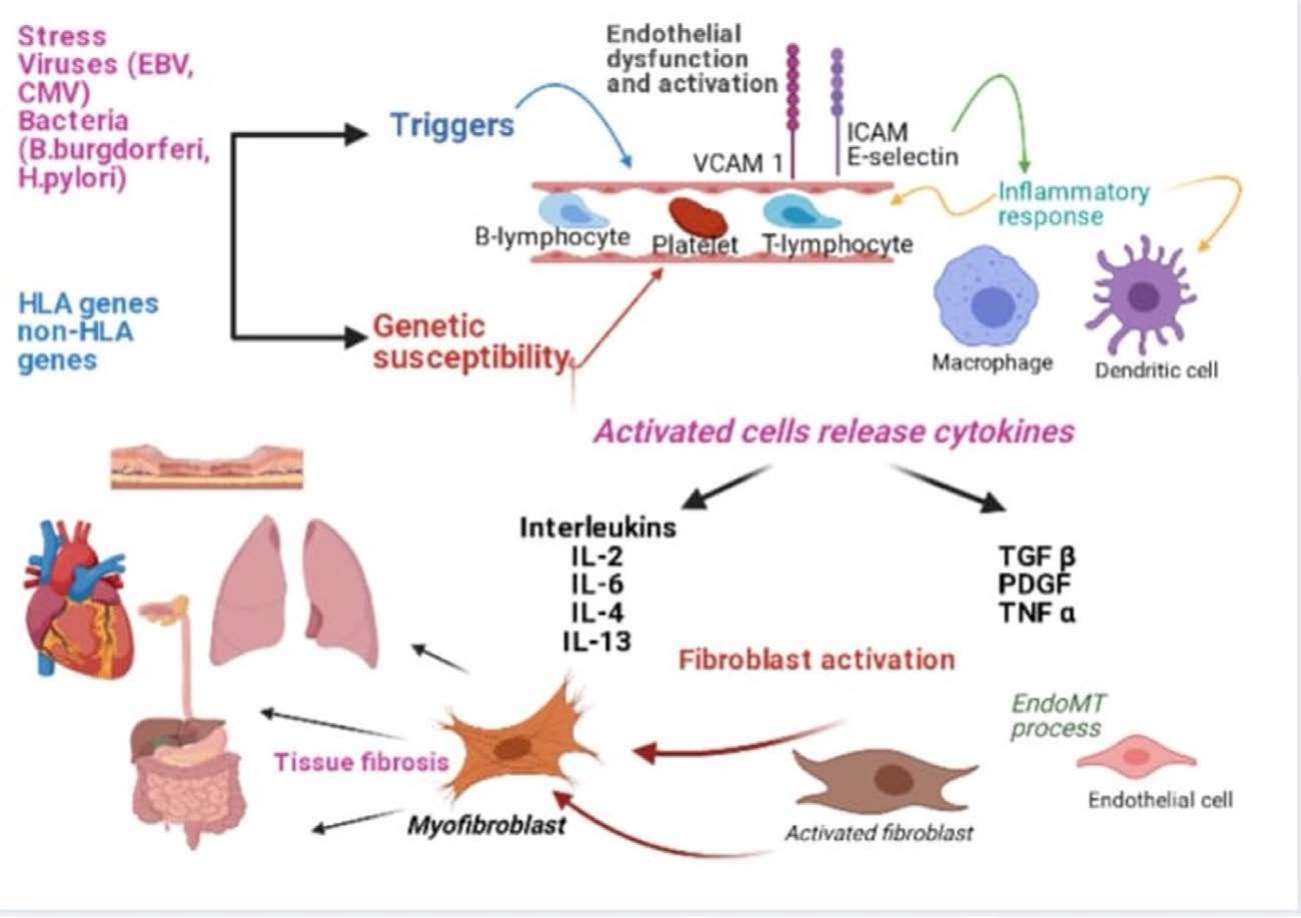
Pathophysiology of systemic sclerosis.

SSc is a complex autoimmune disease that targets connective tissues in the human body’s skin and various internal organs. The predisposing factors for SSc include long-standing primary Raynaud’s phenomenon, stress, silicone breast implants, and infection.^[22–24]^ Together with endogenous triggers, these factors can promote specific epigenetic mechanisms in genetically predisposed individuals. The SSc manifestations progress with microvascular damage triggered by inflammatory responses, activation of innate and adaptive immune systems, generation of autoimmune autoantibodies, and fibroblast activation leading to extensive tissue fibrosis (Fig. 1).^[22,25,26]^

The SSc progressively develops under the impact of endothelial dysfunction and a cascade of events resulting in an imbalance of vasoconstrictor and vasodilator factors. Vasoconstriction in SSc leads to the thickening of the vessel wall and luminal narrowing that eventually reduces the number of capillaries, thereby triggering tissue hypoxia.^[22,27,28]^ The vascular damage predominantly impacts the small and medium-sized vessels of SSc patients’ skin, lungs, heart, kidney, and GI tract.^[23]^

The fibrogenesis process in SSc relies on the overactivation of fibroblasts and loss of their homeostatic state, leading to their trans-differentiation in peripheral tissue into metabolically active myofibroblasts.^[27,29,30]^ The activation of fibroblasts and myofibroblasts contributes to excessive collagen deposition, promoting tissue fibrosis in the skin, lungs, heart, and GI tract of the SSc patients.^[22,29]^

## 4. GI involvement and its management in SSc

### 4.1. Oral cavity

Approximately 68% of patients with SSc experience Sicca syndrome.^[31]^ The labial minor salivary gland biopsy is the recommended prognostic tool to evaluate the risk of lymphoma in patients with systemic scleroderma. In addition, this biopsy helps determine the presence of germinal center lesions (via light microscopy) that impact 5% of SSc patients with a high predisposition for lymphoma.^[32]^

The SSc patients with Sicca syndrome require an intraoral assessment to evaluate their risk for mandibular bone resorption, caries, and periodontitis.^[33]^ Osseous resorption, mainly in muscle attachment areas, such as the mandibular angle and condylar head. Temporomandibular joint impairment may result from condylar resorption, erosive synovitis, musculoskeletal atrophy, and arthritis.^[34]^ In patients with scleroderma, temporomandibular dysfunction reduces oral mobility and opening, resulting in further distension and bloating.^[35]^ These patients further require health education to increase their dental care and salivary secretions to reduce their mouth dryness. However, the treatment strategies include rehabilitation via orofacial exercises and the administration of cevimeline, pilocarpine, muscarinic agonists, and artificial saliva.^[36–38]^

### 4.2. Esophagus

Esophageal involvement occurs almost in all patients with systemic sclerosis.^[39]^ The investigation and management target dysmotility disorder and its manifestations, including dysphagia, gastrointestinal reflux disease, heartburn, and regurgitation. Manometry further helps evaluate dysphagia and its clinical complications. The typical findings include absent peristalsis, ineffective peristalsis, and hypotensive lower esophageal sphincter pressure.^[40,41]^ Recently, high-resolution manometry has been a preferred diagnostic approach due to its potential to detect esophageal dysmotility in asymptomatic patients,^[42]^ despite its controversial role in clinical practice.^[43]^ Esophageal pH monitoring is mainly performed for patients with refractory reflux symptoms or lung transplant candidates.^[44]^

Esophagogastroduodenoscopy (EGD) effectively diagnoses reflux-related esophagitis, esophageal dysmotility, *Helicobacter pylori* (*H. pylori*) infection, esophageal strictures, Barrett esophagus, and adenocarcinoma. A retrospective study performed on asymptomatic patients confirmed reflux esophagitis in 77% of cases of EGD. The study findings also revealed distal esophageal dysmotility in 85% of patients, gastritis in 92% of patients, and *H. pylori* infection in 38% of patients. These findings emphasize the significance of early detection and management in improving the prognostic outcomes in SSc scenarios.^[45]^

The systematic management of GERD and esophageal motility disorder warrants multifaceted approaches relying on lifestyle management.^[46]^ The preliminary measures include head elevation at night, excluding triggering foods/substance abuse and consuming small/frequent meals during the day. Proton pump inhibitors (PPIs) are standalone medications for the clinical management of GERD. PPIs further improve clinical symptoms and prevent esophageal complications in patients with systemic sclerosis.^[47]^ In patients with modest clinical response to daily or twice daily PPIs, the reduction in nighttime reflux symptoms and GERD-associated sleep disturbances in SSc scenarios warrants the administration of PPIs (twice daily) and H2 receptor inhibitors (at bed time).^[48,49]^ Including prokinetic drugs, like cisapride, domperidone, and metoclopramide, with the PPI regimen may further enhance the therapeutic outcomes in SSc patients with esophageal dysmotility or partiality response to PPI management. The therapeutic approaches based on PPIs and prokinetic medications effectively reduce GERD manifestations while improving gastric emptying, esophageal sphincter pressure, and intestinal peristalsis.^[50–52]^ Endoscopic dilatation and continuous administration are preferred therapies for systematically managing and preventing esophageal strictures in systemic sclerosis. EGD surveillance with biopsy is recommended in Barrett’s esophagus, and treatment includes endoscopic ablation or resection of dysplastic epithelium using photochemical, thermal, or radio ablation energy.^[53]^

### 4.3. Stomach

Gastroparesis in SSc leads to early satiety, nausea/vomiting, abdominal pain, bloating, and distention. The assessment of delayed gastric emptying via scintigraphy in SSc settings helps diagnose the onset of gastroparesis. EGD further assists in ruling out gastric outlet obstruction and *H. pylori* infection or gastritis in SSc patients with gastroparesis. The first-line therapy for gastroparesis relies on dietary modifications that necessitate the frequent intake of low-fat/fiber-based diet and vitamin supplementations. The potential of prokinetics in the clinical management of gastroparesis appears questionable in the absence of their safety and efficacy profiles. However, the medical literature supports using metoclopramide (liquid formulation for 12 weeks) for treating gastroparesis.^[54,55]^ Clinical studies also report the adverse effects of metoclopramide following its prolonged use among patients with systemic sclerosis. Other off-label medications with the potential to manage gastroparesis in SSc include domperidone, erythromycin, cisapride, and ghrelin agonists.^[56,57]^ The administration of antiemetics, however, provides symptomatic relief while preventing nausea and vomiting in SSc patients. More invasive procedures, such as a jejunostomy tube or gastrostomy tube for feeding and stomach decompression, may be considered in patients who experience refractory symptoms after receiving pharmacotherapy. A laparoscopic or endoscopic pyloroplasty is rarely performed for managing gastroparesis in SSc scenarios.^[58]^ The medical literature provides weak evidence regarding the role of gastric electrical stimulation in controlling abnormal rhythms, stimulating gastric emptying, and providing symptomatic relief in SSc patients with gastroparesis.^[57,59]^

In patients with symptomatic anemia or suspected occult gastrointestinal bleeding, EGD is the gold standard of care due to its potential to diagnose vascular lesions, small telangiectasias, gastric antral vascular ectasia (GAVE), or so-called “watermelon stomach.” The endoscopic examination helps diagnose GAVE by tracking the longitudinal flat rows from the pylorus to the antrum.^[60]^ The recommended treatment options for GAVE include endoscopic coagulation with laser therapy, argon plasma coagulation, and radiofrequency intervention in refractory cases.^[61]^ However, the treatment of symptomatic anemia in SSc cases relies mainly on intermittent blood transfusions and iron supplementation.

### 4.4. Small intestine

The cross-sectional imaging of the small intestine is the method of choice to evaluate pseudo-obstruction in the abdomen. The cross-sectional imaging assists in evaluating dilatation in the small intestine in the absence of mechanical obstruction. However, the treatment modalities include prokinetics like cisapride,^[62]^ metoclopramide, and domperidone in conjunction with antibiotics.^[63]^ Medical literature also reveals the efficacy of octreotide in improving abdominal symptoms and stimulating intestinal motility in patients with SSc.^[64]^ The gold standard for diagnosing small intestinal bacterial overgrowth (SIBO) relies on the microbial culture of jejunal aspirates. The diagnostic affirmation depends on the finding of >10³ colony factor unit.^[65]^ The hydrogen (glucose or lactulose) and methane breath tests are largely utilized in clinical settings due to their noninvasive nature and easy availability. The positivity of the breath test relies on the hydrogen concentration of ≥ 20 parts per million during the 90 minutes glucose or lactulose challenge and an increase in methane levels to ≥ 10 parts per million.^[65]^ The breath test’s 60% sensitivity and 80% specificity make it a favorable diagnostic option for SIBO assessment.^[66,67]^ The majority of the SSc patients exhibit nutritional deficiency, malnutrition, and weight loss, requiring evidence-based management via nutritional support and hydration.

The symptomatic treatment of SIBO relies on systematic selection and administration of antibiotics.^[68]^ The absence of a standard therapeutic regimen for SIBO warrants its systematic management based on culture findings, the severity of symptoms, and clinical response. Rifaximin is a frequently reported antibiotic in meta-analysis studies demonstrating clinical effectiveness against SIBO in SSc settings.^[68–70]^ Other antibiotics with therapeutic efficacy for SIBO include ciprofloxacin, norfloxacin, amoxicillin, tetracyclines (doxycycline), metronidazole, and trimethoprim-sulfamethoxazole.^[71–73]^ The therapeutic management of SIBO warrants the treatment duration of 10 to 14 days based on the severity of reported symptoms. The meta-analysis by Zhong et al (2017) affirms the therapeutic efficacy of probiotics in alleviating abdominal pain and other intestinal manifestations during SIBO in SSc.^[74]^

### 4.5. Colon and anorectal involvement

Approximately 20% to 50% of patients with SSc report intestinal manifestations based on diarrhea and constipation. Constipation in SSc progresses due to slow intestinal transit based on muscle atrophy and neuropathy. The diagnostic assessment relies on digital rectal exam and computerized tomography or abdominal radiography in patients with colonic pseudo-obstruction, dilatation or impaction, volvulus, megacolon, or perforation. Colonoscopy effectively evaluates the etiology of cancers and lower GI bleeding (telangiectasia) in patients with systemic sclerosis.

The empirical treatment measure for SSc includes administering stimulant laxatives and stool softeners for constipation management. The new secretory agents including, lubiprostone, linaclotide, and Plecanatide, effectively treat chronic constipation.^[75–77]^ However, their safety and efficacy appear questionable in SSc scenarios. Prokinetic agents, including prucalopride, also exhibit therapeutic efficacy for constipation in SSc.^[78]^

Diarrhea in patients with SSc requires multifactorial approaches for its clinical management. The Clostridium difficile is a preferred diagnostic modality to rule out infectious manifestations. The differential diagnoses, including bile acid malabsorption, fructose intolerance, SIBO, and amyloidosis, correlate with persistent colonic inflammation in systemic sclerosis. The treatment guided by etiology effectively challenges the pathology of SSc and improves its prognostic outcomes. The first-line therapy to manage the colonic manifestations in SSc includes dietary modification or targeted dietary therapy based on FODMAP administration (Fermentable Oligosaccharides, Disaccharides, Monosaccharides, and Polyols).^[79,80]^ The selective and cautious use of loperamide also assists in managing diarrhea in patients with SSc.

Nearly 20% to 40% of SSc patients develop fecal incontinence^[81,82]^ that manifests involuntary soiling via recto-anal inhibitory reflex and a decrease in internal anal resting tone.^[83]^ The severity of fecal incontinence in SSc patients substantially deteriorates their health-related quality of life and quality-adjusted life years.^[84]^

Diagnostic modalities performed in individuals with suspected fecal incontinence include anorectal manometry, magnetic resonance defecography, endoanal ultrasound, and balloon expulsion test.^[85]^ The treatment options include dietary modifications, anti-diarrheal medications, and antibiotic treatment in case of concomitant SIBO. Anorectal biofeedback training displays encouraging results in improving symptoms and health-related quality of life.^[86]^ Sacral nerve stimulation is the preferred treatment option in more severe circumstances based on its safety and efficacy in SSc cases.^[87]^

## 5. New treatments and recommendations in GI manifestations in scleroderma

SSc predominantly impacts the GI tract of the affected patients, and the gastrointestinal manifestations reportedly occur in 90% of patients with systemic sclerosis. SSc substantially deteriorates esophageal functioning in most cases; however, it also triggers GERD, esophageal dysmotility, strictures, pancreas, and hepatopancreatic manifestations.^[88]^

Dietary modifications must support the recommended treatment modalities for managing gastrointestinal manifestations in scleroderma cases to improve prognostic outcomes. The dietary modifications (for improving the digestive processes) rely on the administration of probiotics, low-fermentable oligosaccharides, disaccharides, monosaccharides, and polyols (low-FODMAP). The exclusion of smoking habits is further recommended to reduce the risk of clinical adversities.^[89]^ In 2017, the EULAR released the updated recommendations for treating SSc.^[9]^ The experts recommended the administration of PPIs in symptomatic patients with GERD to prevent their gastrointestinal complications. The co-administration of H2 blockers and sucralfate with PPIs in many scenarios assists the management of gastrointestinal complications in SSc. However, the individualization of combination therapies depends on the intensity and severity of the reported gastrointestinal complications. The use of surgical procedures like Nissen fundoplication or the Roux-en-Y can also be considered on a case-to-case basis. PPIs in asymptomatic patients are controversial, given the side effects of long-term PPI use.^[90]^

SSc triggers motility dysfunction in the entire GI tract that warrants the administration of PPIs and prokinetic drugs, including metoclopramide, erythromycin, domperidone, and cisapride. The prokinetic medicines and PPIs effectively increase the lower esophageal sphincter pressure and peristaltic amplitude in the distal esophageal body. The open-label study by French (2018) revealed the therapeutic efficacy of oral 5-hydroxytryptamine 1A (5-HT1A) receptor agonist (Buspirone) in terms of increasing the lower esophageal sphincter resting and reducing the severity of heartburn and regurgitation over 4 weeks.^[85]^

The gastric dysmotility treatment relies on prokinetic drugs administered for esophageal dysmotility. The novel treatment option includes Gastric Per-Oral Endoscopic Pyloromyotomy (G-POEM) that may increase the therapeutic outcomes in SSc patients with failed medical therapy. The treatment goal for managing GAVE includes reducing bleeding and obliteration of ectasia via radiofrequency ablation, laser therapy, or endoscopic coagulation with argon plasma coagulation intervention.^[91]^

The peristaltic dysfunction in the small bowel with associated SIBO eventually leads to malabsorption and malnutrition in patients with systemic sclerosis. The treatment modality relies on administering antibiotics between 14 and 21 days based on the severity of diarrhea and other intestinal complications. Medical literature recommends daily therapy with alternating antibiotics every 2 weeks to minimize the risk of relapsing disease.^[88]^ The clinical studies also recommend the use of probiotics for treating the episodes of abdominal distension and bloating in SSc.^[92]^

The management of constipation in SSc relies on the administration of stool softeners, a high-fiber diet, and probiotics. Prucalopride, a 5HT4 receptor agonist, is a possible therapeutic option based on its potential to improve gastric emptying and proximal colonic motility in systemic scleroderma. Medical literature also supports the therapeutic efficacy of sacral nerve stimulation to improve fecal incontinence and anorectal function in systemic sclerosis. The bulking agents, anti-diarrheal medicines, bile acid-binding resins, and sphincter motor training or biofeedback interventions effectively reduce stool frequency and improve stool consistency in patients with SSc.^[88]^

The liver involvement in SSc is rare and usually related to autoimmune hepatitis and primary biliary cholangitis. Its management depends on prednisone and other medications that antagonize the risk of therapy-induced hepatotoxicity in systemic scleroderma. The development of pancreatic disease in SSc follows the exocrine pancreatic insufficiency requiring enzyme supplementation.^[85,88]^

Cyclophosphamide, Methotrexate, and Mycophenolate Mofetil include the immunosuppressants that effectively treat systemic scleroderma’s vascular, pulmonary, and skin manifestations. The biological medications, anti-fibrotic, and small molecules like Tyrosine kinase inhibitors, rapamycin, and pamidronate also exhibit the therapeutic potential to control the GI manifestations in systemic sclerosis. However, the medical literature does not validate any standard therapy for the medical management of advanced fibrosis in systemic scleroderma. The clinical studies provide limited evidence in favor of the therapeutic efficacy of Intravenous Immunoglobulin for managing gastrointestinal manifestations in systemic sclerosis. The prospective large clinical trials addressing the effect of these novel therapies on the GI tract are needed to optimize the treatment interventions for SSc.^[93]^

## 6. Complications of GI manifestations in scleroderma

The gastrointestinal complications in SSc potentially impact the quality of life and elevate the risk of mortality.^[94]^ Their therapeutic management proves highly challenging in a variety of clinical scenarios.^[94,95]^ The GI complications of scleroderma potentially trigger serious clinical complications that deteriorate the structure and function of the intestine, gastric region, esophagus, oropharynx, and visceral organs. The high prevalence of esophageal and bowel manifestations in SSc substantially deteriorates the affected patients’ quality-assisted life years and survival rate.^[79,95]^

The oropharyngeal complications in SSc emanate from oral and perioral tissue fibrosis, chronic inflammation, histopathological and anatomical changes (due to atrophy), oral cavity disfigurement, and malalignment of osseous structures leading to microstomia and teeth malocclusion. The SSc patients may eventually experience impaired mastication and deglutition, food leakage, regurgitation, voice hoarseness, and aspiration.^[85,96]^ Approximately 1 to 5th of SSc patients experience Secondary Sjogren Syndrome, leading to loss of teeth due to dental caries and periodontal diseases.^[94,97]^

Approximately 50% to 90% of patients with scleroderma experience esophageal manifestations.^[41,85]^ The early identification of esophageal complications in scleroderma cases is necessary to reduce the risk of fatal manifestations. The complications, including organ dysfunction and fibrosis in scleroderma cases, emanate from microvascular changes and inflammatory manifestations in connective tissues, muscles, and nerves. The esophageal complications in SSc potentially reduce esophageal peristalsis and decreasing the lower sphincter pressure. The esophageal manifestations in SSc trigger the development of GERD, esophageal stricture, Barrett’s esophagus, and adenocarcinoma.^[95,98,99]^ The acid reflux further triggers erosive/hemorrhagic esophagitis, leading to esophageal ulcers in patients with systemic scleroderma. The lack of treatment of esophageal complications in SSc patients increases the risk of achalasia-like syndrome, Barrett’s esophagus, and adenocarcinomas.^[100]^ Approximately 12.7% of patients with SSc with 2-years endoscopy status develop Barrett’s esophagus and serious complications.^[101]^ The estimated incidence of Barrett’s esophagus in SSc attributes 6.8% to 12.7% compared to <1% for the general population. The SSc patients with severe symptoms of Barrett’s esophagus experience a high predisposition for adenocarcinoma, esophageal and oropharyngeal cancers than the general population.^[100]^

Approximately 38% to 50% of patients with SSc experience an elevated predisposition for gastric dysmotility that eventually leads to gastroparesis. Severe gastroparesis with persistent nausea and vomiting in SSc cases triggers dehydration and electrolyte abnormalities.^[95]^ GAVE in SSc potentially triggers chronic gastrointestinal bleeding and iron deficiency anemia.^[95,102]^

The intestinal pathology in scleroderma adversely impacts the health-related quality of life and requires multidisciplinary management. The intestinal manifestations in SSc lead to the small bowel and colonic complications. The small bowel dysmotility in SSc patients increases their risk of SIBO. Their intestinal hypomotility further induces lumen dilatation and pseudo-obstruction of the intestine. The rare complication of SSc includes bowel wall necrosis and perforation.^[95]^ Malnutrition in SSc progresses via disrupted digestion and malabsorption. Malnutrition and weight loss in SSc also develop under the impact of environmental and genetic factors. In SSc, decreased oral food intake often triggers nausea, vomiting, dysphagia, and perioral changes. In many scenarios, patients with SSc also develop contractures of fingers that eventually impair their meal preparation and eating activities. The appetite reduction in SSc patients also correlates with their depressive manifestations.^[100,103]^ The malnutrition in SSc adds to the disease severity, poor prognosis, and increased mortality rate.^[104,105]^ Several studies have shown that malnutrition negatively affects the outcome of SSc patients and leads to muscle loss.^[106,107]^ The loss of muscle mass in SSc may be explained by several mechanisms, including endothelial dysfunction, microvascular changes, and altered angiogenesis.^[108–110]^ Low fat-free mass index is a prevalence of 20% to 23% in patients with SSc.^[111,112]^ Skin involvement and reduced muscle mass are associated with SSc in patients.^[112]^ Low muscle mass is associated with the severity of the disease.^[113]^

The colonic and anorectal complications lead to a variety of presentations in SSc. Constipation in SSc is a primary manifestation that indicates colonic involvement and leads to severe complications, including megacolon, ulceration, and volvulus. The fecal incontinence in SSc is a consequence of fibrosis and atrophy of the internal anal sphincter that eventually decreases the resting anorectal pressures. Anal sensory neuropathy plays a pivotal role in fecal incontinence among SSc patients.^[79]^ Fecal incontinence predominantly elevates mood swings and depressive episodes in SSc scenarios.^[95]^ Pneumatosis cystoides intestinalis is a rare complication that develops in patients with systemic sclerosis. The SSc patients with pneumatosis cystoides intestinalis develop gas-filled cysts in the subserosa and submucosa of their small or large intestines. They also report abdominal pain or flatulence and change in bowel habits.^[79,98]^ Table 1 outlines the significant morbidity from commonly reported GI manifestations and/or complications. The limited data based on SSc scenarios attributed to the disease’s rarity, designs of the studies, and literature review restrictions.

**Table 1:** Prevalence, complications, and diagnostic management of GI manifestations in patients with SSc.

Organ involvement	Prevalence	Gastrointestinal manifestations/complications and their prevalence	Diagnosis/Management
Oropharyngeal involvement	10–70%^[114]^	Microstomia: 43%–80%^[19,115]^	1. Regular dental exams.^[98]^
			2. Panoramic radiographic exams to assess for osseous changes.^[98,114]^
		Xerostomia and periodontal disease: 30%–73%^[19,115]^	
		Gingival inflammation/bleeding: 60%–73%^[19,41,98,115]^	3. Good oral hygiene and artificial saliva/lubricants to manage dental and oral/perioral soft tissue pathology.^[98,114]^
		Oropharyngeal dysphagia: 25%^[98,116]^	
			4. Mechanical soft foods, small bolus size, mouth stretching, and even bilateral commissurotomy are treatment options for decreased mouth opening.^[114]^
Esophageal involvement	90%; 30–50% can be asymptomatic^[20,47,88,101]^	GERD 90%^[14,117]^	1. EGD is used for diagnosing esophagitis, Barrett’s, and adenocarcinoma.^[85]^
		Lower esophageal sphincter laxity 37.8–55%^[14,41,117,118]^	
			2. Esophageal manometry is used to evaluate esophageal dysmotility.^[85,114]^
		Esophagitis 60%^[47,101]^	
		Esophageal strictures 41%^[47,101]^	
			2. Barium swallow can be used to detect strictures and their severity.^[85]^
		Barrett’s esophagus 12.7–13%^[88,101]^	
			3. pH monitoring to assess for therapeutic efficacy of PPI in GERD.^[85,114]^
			4. PPI is the mainstem of acid suppression treatment required in scleroderma patients to relieve GERD symptoms and prevent complications.
			5. Lifestyle modification is also recommended (avoiding large and late-night meals).^[98,114]^
Gastric involvement	50%^[20,88]^	Gastroparesis 50%^[20,88]^	1. Gastric emptying study.^[85,98,114]^
		GAVE 5.6–22.3%^[20,88,98]^	
			2. EGD is used to diagnose GAVE if the patient has iron deficiency anemia and for therapeutic purposes like laser photocoagulation or endoscopic band ligation.^[85,98,114]^
		Gastric Bleeding Ectasis 0.6–0.8%^[10,119]^	
		Upper GI Bleeding 3.2%^[10,120]^	
			3. Pro-kinetics (metoclopramide, domperidone) are used for gastroparesis management.^[98]^
Small bowel involvement	40%; 20% can be asymptomatic^[98,105]^	Diarrhea: 27.7–79%^[98,120]^	1. Scintigraphy, capsule endoscopy, MRI/CT enterography may be performed to evaluate small bowel involvement and extent.^[98]^
		Small Intestinal Bacterial Overgrowth 33–50%^[88]^	
		Malabsorption 10–25%^[88]^	
		Small Bowel Pseudo-obstruction: 5.4%^[116]^	
			2. Hydrogen-breath test to assess for SIBO.^[79,85,98]^
		Pneumatosis Cystoides Intestinalis Rare^[79,98]^	
			3. C. Diff testing and stool studies may be indicated in patients with diarrhea.^[85,98]^
			4. Abdominal X-ray and CT abdomen for pseudo-obstruction evaluation.^[85]^
			5. Measurement of fat-soluble vitamins if malabsorption is suspected.^[85]^
			6. Diet modification, probiotics, and antibiotics (fluoroquinolones, metronidazole, tetracycline,
			rifaximin) are treatment options for SIBO.^[98,114]^
Colon involvement	20–50%^[20,88,98]^	Constipation: 9.2–38%^[98,120]^	1.Colonoscopy is recommended for scleroderma patients with new-onset constipation.^[79,85,98]^
		Megacolon: 1.5–3.8%^[120]^	
		Large intestine vascular ectasia 1.3–3.1%^[98,120]^	
			2. Fiber supplementation, bowel training, stool softeners, laxatives, and prokinetics are recommended to manage constipation.^[79,98,114]^
		Lower GI bleeding 2.9%^[10,120]^	
		Wide-mouth diverticula 1.3–8.6%^[16,98,120,121]^	
Anorectal involvement	50–70%^[47,79,98,101]^	Rectal Prolapse 20%^[121,122]^	1. Anorectal manometry, MR defecography, and balloon expulsion test are used to assess fecal incontinence.^[79,85]^
		Fecal Incontinence 20–38%^[20,88,98,120,123]^	
		Fecal Impaction 18%^[98,120]^	
			2. Anti-diarrheal agents and diet changes are used to improve stool consistency^[98]^
			3. Sacral nerve stimulation was shown to be beneficial in the management of fecal incontinence.^[79,98,114]^
			4. Surgical intervention may be indicated for rectal prolapse.^[79,114]^
Liver involvement	1.1–1.5%^[98]^	Primary biliary cirrhosis (PBC) 2–18%^[119,123]^	1. Checking liver enzymes, bilirubin, and antimitochondrial antibodies.^[98,114]^
		Autoimmune hepatitis^[85,98]^	
			2. If PBC is suspected but AMA negative, anti-gp210 and anti-sp100 are highly specific.^[98]^
			3. Liver ultrasound if the tests are abnormal.^[114]^
			4. Avoidance of hepatotoxic medications.
			5. Hepatic dosing of medications metabolized by the liver^[85]^
			6. Ursodeoxycholic acid for PBC.^[85,98,114]^

CT = computerized tomography, EGD = esophagogastroduodenoscopy, GAVE = gastric antral vascular ectasia, GERD = gastroesophageal reflux disease, GI = gastrointestinal tract, MRI = magnetic resonance imaging, PBC = primary biliary cirrhosis, PPI = Proton pump inhibitors, SIBO = small intestinal bacterial overgrowth.

## Author contributions

**Conceptualization:** Mahmoud Nassar.

**Data curation:** Victoria Ghernautan, Nso Nso, Akwe Nyabera, Francisco Cuevas Castillo, Wan Tu, Luis Medina, Camelia Ciobanu, Mostafa Alfishawy, Vincent Rizzo, Saphwat Eskaros, Mohamed Khalifa.

**Project administration:** Mahmoud Nassar.

**Supervision:** Mahmoud Nassar, Mamdouh Mahdi.

**Writing – original draft:** Mahmoud Nassar, Victoria Ghernautan, Nso Nso, Akwe Nyabera, Francisco Cuevas Castillo, Wan Tu, Luis Medina, Camelia Ciobanu, Mostafa Alfishawy, Vincent Rizzo, Saphwat Eskaros.

**Writing – review & editing:** Mahmoud Nassar, Victoria Ghernautan, Nso Nso, Akwe Nyabera, Francisco Cuevas Castillo, Wan Tu, Luis Medina, Camelia Ciobanu, Mostafa Alfishawy, Vincent Rizzo, Saphwat Eskaros, Mamdouh Mahdi, Mohamed Khalifa, Mohamed El-Kassas.
